# Electrochemical deposition of layered copper thin films based on the diffusion limited aggregation

**DOI:** 10.1038/srep34779

**Published:** 2016-10-13

**Authors:** Chenhuinan Wei, Guoxing Wu, Sanjun Yang, Qiming Liu

**Affiliations:** 1 School of Physics and Technology, Key Laboratory of Ariticial Micro- and Nano-structures of Ministry of Education, Wuhan University, Wuhan 430072, China

## Abstract

In this work layered copper films with smooth surface were successfully fabricated onto ITO substrate by electrochemical deposition (ECD) and the thickness of the films was nearly 60 nm. The resulting films were characterized by SEM, TEM, AFM, XPS, and XRD. We have investigated the effects of potential and the concentration of additives and found that 2D dendritic-like growth process leaded the formation of films. A suitable growth mechanism based on diffusion limited aggregation (DLA) mechanism for the copper films formation is presented, which are meaningful for further designing homogeneous and functional films.

In the electronic industry, copper films have attract great attention to fabricate the printed circuit boards and electric lead in narrow bezel touchscreen owing to their high electrical conductivity^1^,^2^. Physical vapor deposition (PVD)^3^, chemical vapor deposition (CVD)^4^, magnetron sputtering^5^ and electrochemical deposition (ECD) can be used to prepare. Among these methods, ECD as a green approach has gained extensive attention in recent years, it’s widely accepted that ECD is one of the most extensively used ways due to the cost-effective, time saving and facile technique. During the process of ECD, there are lots of alterable parameters can affect the nucleation and growth process of crystals, such as potential, current, additive, temperature and pH^6^,^7^,^8^.

Additives can partly improve the final shape of deposits. Polyethylene glycol (PEG), polypropylene glycol (PPG) and chloride ions are typical suppressor additives of copper-sulfate-based plating baths. Gu, C. D. *et al.* deposited Cu films with absence and presence of additive of ethylene diamine (EDA), the thickness of films decreased from 11.5 μm to 7.7 μm own to the presence of EDA^1^. Han, Y. J. *et al.* used organic additive malachite green to deposite copper films with square pyramidal crystallites on surface^7^. Based on the superb complexing ability of additive of sodium citrate to chelate copper ions, adsorb phase on Cu surface and transport hindering role for upcoming Cu ions, Whang, T. J. *et al.* prepared homogeneous densely-packed grains and CuInSe_2_ thin films with higher crystallinity^6^. However, these prepared films by ECD may not thin enough and have complex morphology on surface.

Dendritic growth along with other two basic crystal growth mechanisms, spiral growth at screw dislocations (BCF theory)^9^, layer-by-layer growth (LBL)^10^, are the basic crystal growth patterns of metal nanocrystals (NCs). Diffusion limited aggregation (DLA), as a kind of dendritic growth pattern, is usually used to explain phenomena for the formation of fractal in the nature, such as fingerprint of viscosity liquid miscibility^11^, fractal growth of copper^12^, MBE grow crystals^13^ and dendrite growth forms^14^. These DLA growth have observed mostly for Ag, Pt, Ni, Zn^15^,^16^,^17^. DLA theory has been widely researched since it proposed, but using this microscopic theory to explain the physical mechanism of growth has not been generally accepted. Different from recent researches about the screw dislocations growth of nanoplates of zinc oxide (ZnO)_6_, α-Co(OH)_2_, Ni(OH)_2_, and gold, which have highly symmetric structures^18^. Particle attachment is easily observed among small particles due to their larger surface-to-volume ratio and higher collision frequency associated with their greater mobility. A strong thermodynamic driving force impels particle attachment to eliminate surface energy.

In this paper, to our knowledge, we firstly fabricated 57 nm layered Cu films with smooth surface by ECD with sodium citrate as additive. Besides, we introduced a nanomaterial growth based on DLA mechanism to describe the formation process of 2D Cu films by single NCs on ITO substrate.

## Result and Discussion

### Films characterization

**Figure 1:**
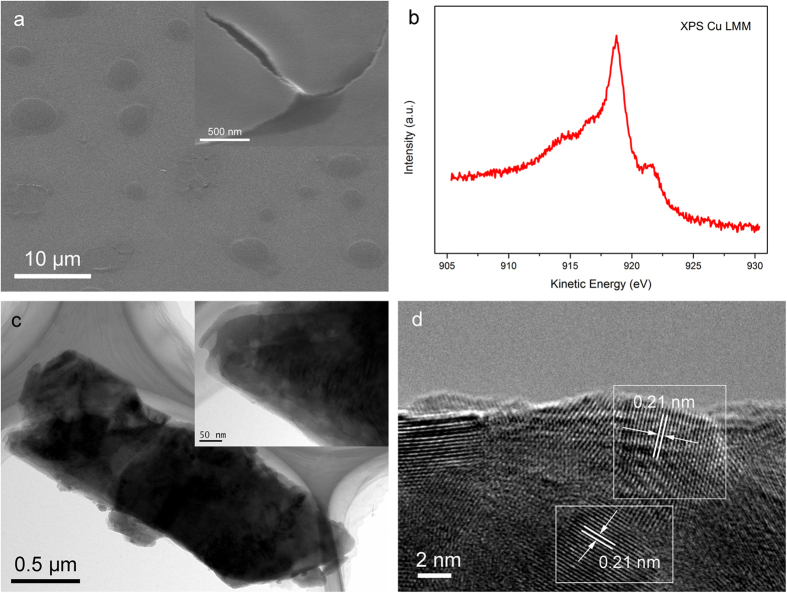
(**a**) SEM images, (**b**) XAES Cu LMM spectra, (**c**) TEM micrograph and (**d**) HRTEM image of the layered copper thin films.

Films were deposited in 2 mM CuSO_4_-based electrolyte solution with 10 mM sodium citrate at −0.3 V vs. Ag|AgCl for 250s. From the SEM image (Fig. 1a), the films comprise nanosheets and connective nanoparticles. The thickness of the films measured by ellipsometer was 56.78 nm. Due to the lattice mismatch of copper nanosheets and indium tin oxide, these nanosheets warps (top right insert of Fig. 1a).

The Cu LMM peak occurs at a kinetic energy of 918.7 eV, which is agreement with the literature values for XAES Cu energy of metallic copper, indicating the composition of the films is Cu metal without oxidation^19^. The distance between neighboring planes was measured to be 0.210 nm in the HRTEM image (Fig. 1d), which is well in accordance with the standard value (2.088 Å) of the (111) lattice planes of Cu crystals (JCPDS#04-0836) with face-centered cubic structure.

Structure characterization of the sheets by TEM (Fig. 1c) illustrate that nanosheets have irregular boundaries with hierarchical organization. A huge nanosheets was folded inward when we separated films from the substrate. It is clear that a few layers fold up together (top right inset of Fig. 1c). In conclusion, above analysis indicate that the sample we prepared by ECD is layered copper films.

**Figure 2:**
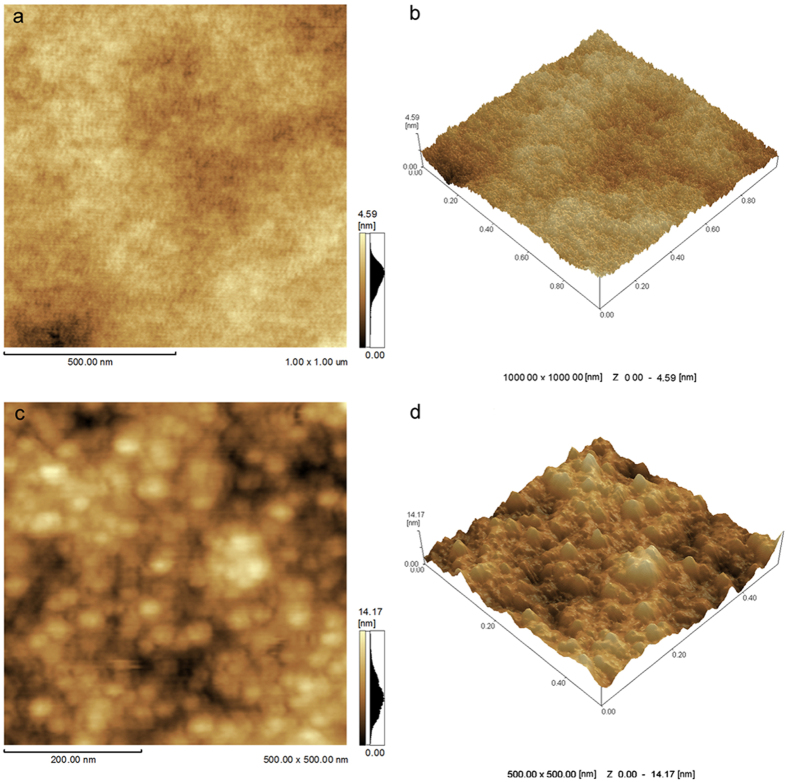
(**a**) 2D and (**b**) 3D AFM micrographs (5 μm × 5 μm) of Cu nanosheets. (**c**) 2D and (**d**) 3D AFM micrographs (500 nm × 500 nm) of nanoparticle films outside sheets.

Figure 2a,b shows the 2D and 3D AFM micrographs of the sheets and the root mean square roughness (Rms) of the sheets is 0.520 nm. Figure 2c,d are the 2D and 3D AFM micrographs of the nanoparticle films outside the nanosheets and Rms is 2.173 nm. These further indicate that films comprise sheets and nanoparticles and the sheets have smooth surface.

### Growth mechanism

The formation of complexing constant of citrate ions and copper ions is 1 × 10^18^, which illustrates that tridentate sodium citrate is a good complexing agent for chelating copper ions^20^ and affect the shape of nanostructures. Besides, the aggregation of particles depend strongly on the balance between the nucleation and growth kinetics, which are both potential dependent^8^. Accordingly, to elucidate the growth mechanism of Cu films, we decided to investigate the effect of sodium citrate and potential on deposits.

**Figure 3:**
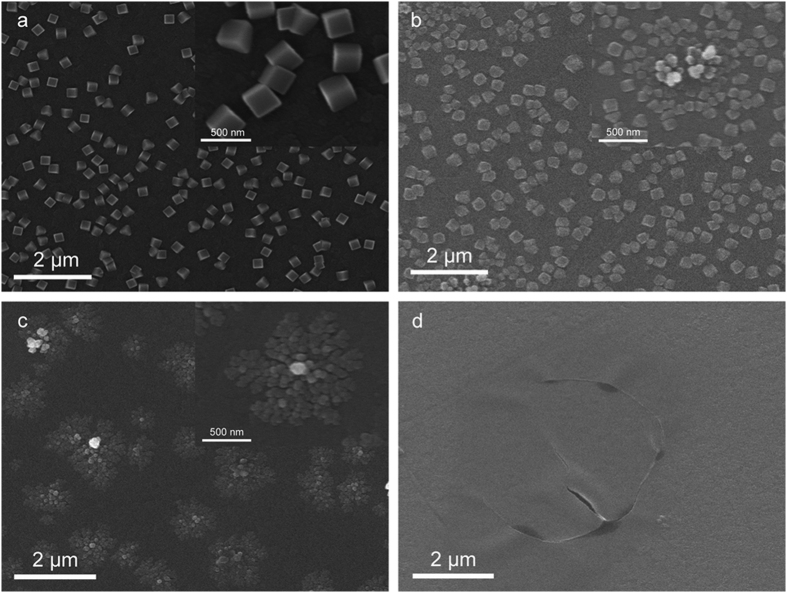
SEM images of copper nanostructures on ITO working electrode at −0.3 V vs. Ag|AgCl for 250s from a 2 mM CuSO_4_-based electrolyte solution with different concentration of sodium citrate: (**a**) 0 mM, (**b**) 1 mM, (**c**) 5 mM and (**d**) 10 mM.

**Figure 4:**
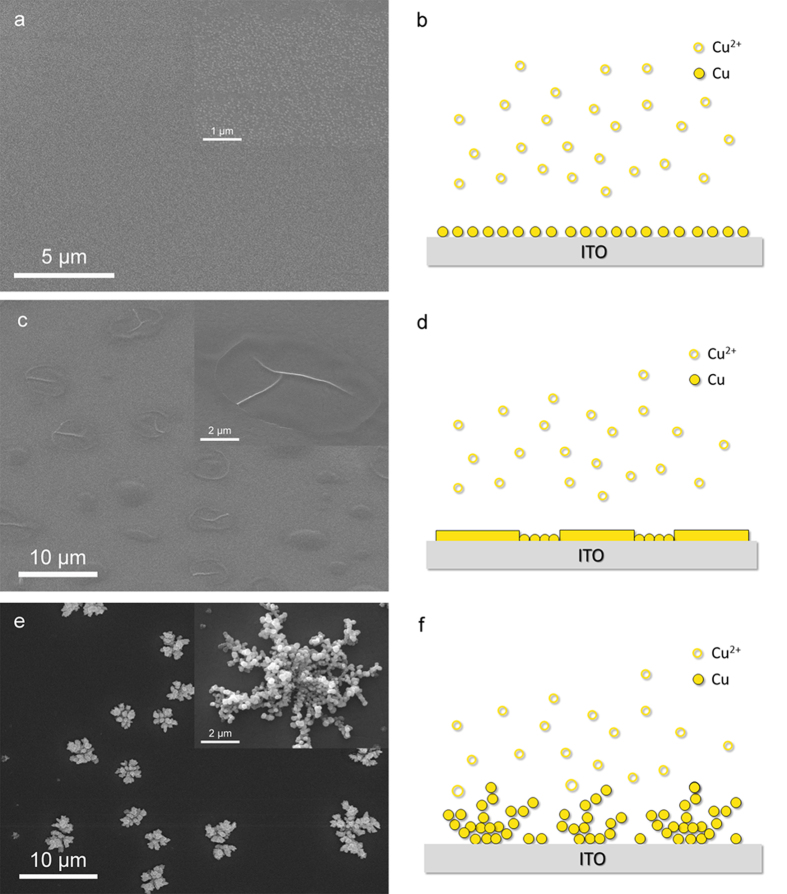
SEM images of copper nanostructures on ITO working electrode from a 2 mM CuSO_4_-based electrolyte solution with 10 mM sodium citrate for 250s at the potential of (**a,b**) −0.25 V, (**c,d**) −0.3 V, (**e,f**) −0.5 V vs. Ag|AgCl.

As observed from Fig. 3, different concentrations of sodium citrate give rise to the formation of remarkably different forms of copper nanostructures. In Fig. 3a, copper microcubes with uniform shape and size distribute randomly on the ITO substrate with absence of sodium citrate in CuSO_4_-based electrolyte solution^21^. With the concentration of sodium citrate raise to 1 mM (Fig. 3b), apex of cubes become unapparent and some of them aggregate together, it indicate that growing up was suppressive during the aggregation process. When the concentration of sodium citrate raise to 5 mM (Fig. 3c), crystals with spherical shape aggregate parallel to the substrate and have a tendency to adapt 2D dendrite growth as complexation enhance. With the concentration reaching 10 mM (Fig. 4d), plane-dendritic crystals turn to nanosheets with smooth surface.

A dramatic change can be observed on the morphology of copper nanostructures as presented in Fig. 4. Figure 4b,d,f are the demonstration figures respond to the upper nanostructures. At the potential of −0.25 V (Fig. 4a,b), individual copper nanoparticles are uniformly deposited on the substrate, kinetic control the nucleation of copper^22^. At the potential of −0.3 V (Fig. 4c,d), growth of copper is weak limited and the process of nucleation is mixed kinetic/diffusion control. The resulting copper nanoparticles ‘attached oriented’, aggregate compactly and extend parallel to substrate to form films. At the more negative potential of −0.5 V (Fig. 4e,f), diffusion is dominant, a Cu dendrite consists of a main stem with long side branches decorated fully by small nanoparticles, the length of the branch can reach 7 μm. Growth instabilities cause reduction of Cu^2+^ at protuberances and urge them to grow branches along preferential directions to form dendrites^23^.

**Figure 5:**
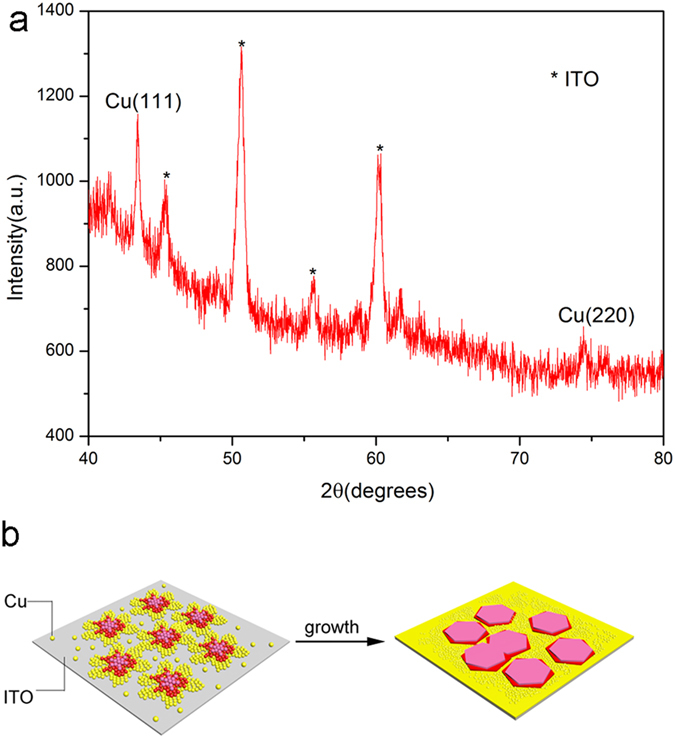
(**a**) XRD patterns of Cu films deposited on ITO substrate. (**b**) Schematic presentation of the ECD process of the copper films.

Combined with our experiments, both additive and potential affected the final shape of copper, and more specifically, they affected the growth and diffusion of copper crystals. Sodium citrate, as a suitable complexing agent shows great ability to control the growth of copper nanostructures. Accordingly, during the process of ECD, sodium citrate molecules can not only chelate with Cu^2+^ but also adjust and improve congeal of nannoparticles to 2D structrures such as silver seed nanoparticles to nanoprisms^24^. The observed X-ray diffraction peaks can be indexed to Cu (JCPDS#04-0836) or ITO as shown in Fig. 5a. The peak of XRD pattern illustrate the surface of the film consists of Cu (111) facets, which possibly attribute to the preferential adsorption of sodium citrate on the Cu (111) surface. This intense adsorption will hinder further reduction of copper ions onto Cu (111) facets and promote the lateral growth of the film, result in 2D copper thin films with smooth surface. This growth is consistent with the observations of Yunyu Joseph Han *et al.*, who used organic additive malachite green (MG) in Cu ECD on Au substrate to form square pyramidal crystallites with Cu (200) direction. They thought the reason was the interactions of MG with Cu (111) surface were stronger than those with Cu (200) surface, and this stronger interaction partly blocked epitaxial growth on Cu (111) surface and prompted further growth on Cu (200) surface^7^.

Relying on the TEM image of layered nanosheets in Fig. 1c and layered dendrites in Fig. 3c, we speculated a possible diffusion limited aggregation mechanism to describe the growth process of the nanosheets. When the voltage is at high stage, seeds of initial structures form firstly, with fast reduction reaction of Cu^2+^, numbers of new copper ions constantly deposit on the ITO substrate, during this time, diffusion is the driving force that controls the dynamics of aggregation of copper nanoparticles to form 2D dendritic nanostructures, this phenomenon could be explained within the diffusion limited aggregation. When kinetic control is prevailing at low voltage, nanoparticles reduce at the edge of plane-dendritic and gradually extend outside. These nanoparticles integrate directional under the influence of citrate ions surfactant, with the expansions of nanoparticles between dendrites during diffusion-limited aggregation, dendrites become dense and connected together, form nanoparticle films outside finally. These nanosheets overlap with prolonged time of ECD. A formation mechanism can be proposed (Fig. 5b).

## Conclusion

In summary, by means of electrochemical deposition, the optimal condition to prepared Cu films was to use sodium citrate as additive in the CuSO_4_-based electrolyte solution with −0.3 V vs. Ag|AgCl for 250s. The 57 nm films consisted of nanosheets and nanoparticles, it had lamellar structure and smooth surface. The effect of sodium citrate and potential on final shape was deeply. Use of sodium citrate as additive resulted in the deposits transferred from nanocubes to films. As potentials become more negative, deposits transferred from particles to films, or even to dendrites. By comparing these phenomena, a suitable growth mechanism based on diffusion limited aggregation can explain the formation of thin films.

## Methods

### Fabrication of Cu thin films

#### Preparation of ITO substrate

Cu films were deposited on ITO substrate (Zhuhai Kaivo Optoelectronic Technology Co. Ltd, sheet resistance is about 10 Ω/□) and the area of immerged part of ITO was about 0.8 cm^2^ (0.8 cm × 1 cm). ITO glasses were cleaned sequentially in an ultrasonic bath with acetone, ethanol and deionized water mixed ammonia water for 5 min, then put into the electric thermostatic drying oven and dried at 50 °C for several minutes before ECD.

#### Copper thin films deposition

The ECD of Cu thin films were conducted in a 20 ml electrolyte solution including 2 mM CuSO_4_·5H_2_O, 10 mM C_6_H_5_Na_3_O_7_·2H_2_O, 1.5 ml n-propanol and deionized water. A electrochemical workstation (CHI760D, ShangHai Chenhua) interfaced with a computer to a typical three-electrode cell containing a Ag|AgCl reference electrode (RE), a platinum counter electrode (CE) and a indium-doped tin oxide (ITO) glasses working electrode (WE). Multi-potential step (STEP) was applied to obtain the copper thin films from the mixture solution. At early stage of STEP, the forward (VF) and reverse (VR) potentials were −1 V vs. Ag|AgCl (100 ms) and −0.35 V vs. Ag|AgCl (50 ms), respectively. Then the constant voltage was −0.3 V vs. Ag|AgCl to deposit films. The deposition time lasted 250s. All experiments were performed under constant room temperature conditions.

### Materials characterizations

Scanning Electron Microscope (SEM) analysis was performed on a FEI Sirion FEG with an accelerating voltage of 20 kV. Transmission electron microscope (TEM) analysis was performed on a JEM-2100 transmission electron microscope with an accelerating voltage of 200 kV, while high resolution transmission electron microscopy (HRTEM) in lattice fringe mode was performed in the samples using a JEM2012–FEF electron microscope (JEOL, Japan) operating at 200 kV. The thickness of the films was characterized by ellipsometer (Rudolf Research/Auto El-II). The chemical species of the films were tested by X-ray photoelectron spectroscope (XPS, ESCALAB 250Xi, Thermo Fisher Scientific, USA) using Al Kα radiation of 1486.6 eV as the excitation source. Surface morphology of copper thin films was characterized by atomic force microscopy (AFM, SPM-9500J3, Shimadzu) in contact mode technique.

## Additional Information

**How to cite this article**: Wei, C. *et al.* Electrochemical deposition of layered copper thin films based on the diffusion limited aggregation. *Sci. Rep.* **6**, 34779; doi: 10.1038/srep34779 (2016).
